# Risk assessment of manual handling operations at work with the key indicator method (KIM-MHO) — determination of criterion validity regarding the prevalence of musculoskeletal symptoms and clinical conditions within a cross-sectional study

**DOI:** 10.1186/s12891-017-1542-0

**Published:** 2017-05-10

**Authors:** Andre Klussmann, Falk Liebers, Hansjürgen Gebhardt, Monika A. Rieger, Ute Latza, Ulf Steinberg

**Affiliations:** 1 Institute of Occupational Health, Safety and Ergonomics (ASER), Corneliusstrasse 31, D-42329 Wuppertal, Germany; 2 Federal Institute for Occupational Safety and Health (BAuA), Nöldnerstrasse 40-42, D-10317 Berlin, Germany; 3 Institute of Occupational and Social Medicine and Health Services Research, University Hospital of Tübingen, Wilhelmstrasse 27, D-72074 Tübingen, Germany; 4 University of Wuppertal, School of Mechanical Engineering and Safety Engineering, Chair of Human Engineering, Gaussstr. 20, D-42119 Wuppertal, Germany

**Keywords:** Risk assessment, Manual handling operations, Cross-sectional study, Musculoskeletal symptoms, Criterion validity, WRULDs

## Abstract

**Background:**

Manual handling operations (MHO) are known to be risk factors for work-related upper limb disorders (WRULDs), e.g. symptoms and conditions such as carpal tunnel syndrome. To estimate the risk of WRULDs, a Key Indicator Method (KIM) for the risk assessment of MHO was developed. The method was validated in regard to different criteria, including face validity, criterion validity, reliability and further aspects concerning utility. This paper describes the KIM-MHO and criterion validity of this method with reference to prevalence of musculoskeletal disorders (MSDs).

**Methods:**

A cross-sectional sample of 643 employees exposed to MHO was compared to a reference group of 804 unexposed subjects predominantly working at visual display terminals. The Nordic Questionnaire and a standardized clinical examination were used to obtain the 7-day and 12-months prevalence of symptoms and clinical conditions of the musculoskeletal system. Job specific exposure levels to MHO were assessed by ergonomists using the KIM-MHO. The resulting risk scores were categorized into risk categories 1 - low risk (reference group), 2 - increased risk, 3 - highly increased risk, and 4 - high risk. Log-linear Poisson regression models were applied to obtain adjusted prevalence ratios (PR) with 95%-confidence intervals.

**Results:**

The 7-day prevalence of symptoms for subjects in risk category 3 compared to risk category 1 was significant for the regions shoulder [women (w): PR 1.8 (1.2–2.7), men (m): PR 2.3 (1.2–4.4)], elbow [w: PR 3.3 (1.5–7.2), m: PR 2.4 (0.8–7.3)], and hand/wrist [w: PR 3.0 (1.7–5.3), m: PR 5.5 (2.7–11.3)]. The 7-day prevalence of symptoms for risk category 4 was also significant for the regions shoulder [w: PR 1.9 (1.3–2.8), m: PR 1.9 (1.3–2.7)], elbow [w: PR 4.5 (2.3–8.7), m: PR 3.3 (2.1–5.4)], and hand/wrist [w: PR 4.2 (2.6–6.9), m: PR 5.5 (3.5–8.5)]. The 12-months prevalence in these joint regions show comparable increases in the risk categories 3 and 4.

**Conclusions:**

The KIM-MHO is valid in regard to criterion validity. The hypothesis could be confirmed, that high risk scores were associated with an increased prevalence of symptoms and clinical conditions especially in the shoulder, elbow and hand/wrist regions among employees exposed to MHO.

**Electronic supplementary material:**

The online version of this article (doi:10.1186/s12891-017-1542-0) contains supplementary material, which is available to authorized users.

## Background

Symptoms and disorders of the musculoskeletal system (MSDs) are the most common reasons for absence from work in Germany [1]. 18.9 million days of sick leave were caused by symptoms and disorders of the musculoskeletal system and connective tissue in 2015. This is equivalent to 26.8% of all days off work [2–4]. The resulting economic loss amounted to 19.2 billion euros in 2011 [1]. Upper extremity musculoskeletal disorders are still common in the working population [5–7], for example carpal tunnel syndrome (CTS) [8], and medial or lateral epicondylitis [9].

In addition to manual handling of heavy loads, exerting high forces, working in awkward postures and repetitive hand/arm movements are the most frequently discussed work-related physical risk factors [10, 11]. Many different occupational groups are associated with CTS, e.g. in manufacturing, fishing or construction. Those at risk include workers on assembly lines, meat packers, poultry workers, gardeners, musicians, farmers, mechanics and factory workers [12].

According to the European Council Directive 89/391/EEC of 12th June 1989 on the introduction of measures to encourage improvements in occupational safety and health (OSH), the employer must perform an assessment of the risks to safety and health at work, including those to which specific groups of workers are exposed [13]. Long-lasting, dynamic repetitive exposure of the hand-arm system during manual handling operations (MHO) alone or in combination with static and postural effort are known as causes of musculoskeletal symptoms and disorders [14–19]. But based on a systematic review, da Costa et al. claimed, that studies with an adequate methodology designed to clarify the incidence and prevalence of WRMSDs are sparse and raise concerns in regard to the causal relationship [16]. According to the BIBB/BAuA Employment Survey in 2012, based on interviews of a random sample of 20,000 employees in Germany, 42.5% of the employees were exposed to MHO, and 18.2% suffered complaints from this exposure [20]. An ergonomic design and organization of workplaces should focus on reducing harmful exposures to the musculoskeletal system and thus the associated symptoms and disorders. Therefore, the assessment of manual work tasks is crucial to estimate health risks of exposed employees and to implement conditional prevention strategies in the working area to reduce such exposures.

Currently, a number of risk assessment methods for the evaluation of work-related physical exposures exist, e.g. Hand-Arm-Risk-Assessment Method (HARM) [21], Manual Tasks Risk Assessment Tool (ManTRA) [22], Assessment of Repetitive Tasks of the upper limbs (ART) [23], Job Strain Index (SI) [24], American Conference of Governmental Industrial Hygienists Threshold Limit Value for Mono-Tasks Handwork Hand Activity Level (ACGIH TLV-HAL) [25]. Takala et al. [26] analyzed existing methods systematically according to concurrent and criterion validity, repeatability, and aspects related to utility. However, the authors stated that many of these methods underwent only insufficient validation processes [26]. This is a relevant limitation, since the explanatory power of the method is not clear. What kind of risk (e.g. pain, accidents, job satisfaction, or fatigue) is assessed and what does the result mean? In addition, validated assessment tools are one prerequisite for the development and implementation of evidence-based OSH measures [27]. Only the SI and the HAL were tested against all quality criteria indicated above. Both methods are widely used internationally. The SI has the disadvantage that the data on frequencies of movements and force levels are assessed relatively broadly. In the case of the HAL, no activities lasting less than 4 h could be considered.

A new method for the assessment of working conditions involving manual handling operations was developed by the Federal Institute for Occupational Safety and Health (BAuA) and released as a draft in the year 2007 [28]. This draft of the so-called Key Indicator Method for Manual Handling Operations (KIM-MHO) was developed analogous to the existing KIMs for Lifting, Holding and Carrying (KIM-LHC) and for Pulling and Pushing (KIM-PP) of loads [29]. The draft of KIM-MHO was evaluated and modifications for its improvement were made [30]. Considering the requirements mentioned above, the method was validated regarding different criteria, including face validity, criterion validity, reliability and further aspects related to utility. The revised KIM-MHO and the results of the validation in regard to criterion validity are described in this article. An advantage of the KIM-MHO compared to the other methods mentioned above is that the force exertions can be considered more differentially. Furthermore, the method is designed to be applicable for any duration of an activity. Thus, even minor improvement measures (e.g. reducing the force level of individual motions) or elimination of unnecessary motions can be visualized and utilized for implementation of even minor improvements.

## Methods

### Aim and study design

The aim of the study is to determine the criterion-related validity by comparing the results of the KIM-MHO with the prevalence of musculoskeletal symptoms in employees within a cross-sectional study design in terms of concurrent validity. It is assumed, that employees at workplaces with high exposures to MHO show adverse health-related outcomes (musculoskeletal symptoms) more frequently than non-exposed workers. Concordantly, it is assumed as a hypothesis regarding criterion validity, that an assignment of workplaces to high risk categories by KIM-MHO is correlated with an increased frequency of musculoskeletal symptoms in exposed workers compared to unexposed workers. Relevant confounders such as age and gender are taken into consideration. In contrast, the KIM-MHO should result in low scores at workplaces with low exposures to MHO and low frequencies of musculoskeletal symptoms in workers. Therefore, the validity of KIM-MHO is rated as satisfactory, if a dose-response relationship for the most affected body regions of the upper extremities is observed. For the analysis of criterion validity, the recommendations of the COSMIN checklist (COnsensus-based Standards for the selection of health Measurement INstruments) are taken into account [31].

### Setting of the study and characteristics of participants

A survey was carried out among employees exposed to MHO. A total of 642 employees (207 women, 435 men) with exposures to MHO at 17 different workplaces in Germany were included. Basic inclusion criteria for the selection of companies were: workplaces with homogeneous manual work most of the shift, and consent of management and workers’ council. Cooperative companies in previous projects that fulfilled the inclusion criteria, were addressed specifically.

Male and female employees 18 to 65 years of age at workplaces with MHO at different levels of exposures were recruited, who worked at the workplaces studied for at least 3 months and were proficient in the German language. The aim was to cover all employees of the selected workplaces. If more than 40 employees were engaged at the same workplace, the participants were selected randomly.

These exposed employees were compared with a reference data set of 804 employees (306 women, 498 men) working at well-designed visual display terminal (VDT) workstations in a large German enterprise. This reference data set was generated in a former cross-sectional study using a similar methodology of data collection [11]. In both cross-sectional studies, all available male and female employees at the selected workplaces were asked to participate voluntarily (inclusion criterion).

### General description and application of the KIM-MHO

For what kind of work activity this method can be applied?

- The KIM-MHO serves to assess work activities predominantly involving exposure to the finger-hand-arm area when working on physical objects (manual jobs). Typical indicators of these work activities are frequent repetitions of identical or similar manual operations and requirements regarding dexterity or the recognition of small details.

Work activity where this method cannot be applied include:

- Activities involving the manual handling of loads (transport of loads with weights in excess of 5 kg). For these activities, the use of the two other Key Indicator Methods (KIM-LHC and KIM-PP) is recommended.
- Activities involving high energy requirements due to whole-body work and high exertion of action forces (e.g. rising, climbing, machine assembly).
- Activities involving long-duration forced postures (e.g. kneeling, bending, and lying).

What are the key indicators of manual handling operations used in KIM-MHO?

The key indicators (KI) considered in the KIM-MHO are:

1. Total duration of manual handling operations per shift,
2. Type, duration and frequency of force exertion(s),
3. Force transfer/gripping conditions,
4. Hand-arm position and movement,
5. Work organization (e.g. variation of exposures),
6. Working conditions (e.g. noise, climatic conditions), and
7. Body posture.

What is the result of the risk assessment?

The different key indicators are classified in different scales. By multiplying the scale value of the daily duration of the activity (KI 1) with the sum of the other scale scores (KI 2 to KI 7), a total value can be calculated. This score can be allocated to one of four risk categories, which are characterized by increasing intensity of manual handling operations and increasing risk estimation of overload and work-related adverse health effects:

- Risk category 1 (<10 points, “green”): low exposure situation where physical overload due to MHO is unlikely to occur,
- Risk category 2 (10 – 24 points, “greenish yellow”): increased exposure situation, physical overload due to MHO is possible for particular groups of employees. Redesign of the workplace might be helpful especially for this group,
- Risk category 3 (25 – 49 points, “yellow”): highly increased exposure situation, physical overload due to MHO quite possible. Redesign of the workplace is recommended,
- Risk category 4 (≥ 50 points, “red”): high exposure situation, physical overload due to MHO is likely to occur. Redesign of the workplace is necessary.

The worksheet of the KIM-MHO including a short manual, explaining how the assessment is to be performed is given in the Additional file 1. An additional interactive worksheet and an extended user manual are available [32].

### Application of the KIM in this study

Metadata of the workplace (such as name and type of activity, typical working time and number of repetitions per shift, distribution of the work task during the shift, description of other relevant exposures such as noise level or lighting) were documented in an ergonomic work procedure analysis. A video documentation was done for every workplace. Based on this work place analysis, the KIM-MHO was completed by two occupational scientists (AK and US) to determine the criterion validity. In case of divergent results, the categorizations were discussed and a consensus found.

For the determination of objectivity and reliability, the methods were also applied by the local practitioners (e.g. industrial engineers, safety engineers, occupational health physicians, ergonomist) and a group of practitioners from other companies. The results of these tests are not covered by this article, but are documented in the research report [33].

### Exposure variable

The four risk categories based on the score value of the KIM-MHO were considered as independent variable. Subjects in risk category < 10 points according to KIM-MHO were considered as reference group.

### Outcome variables

Methods of the survey and of the physical examination including diagnostic criteria are available in the previously published study protocol [34].

Physical examinations of the exposed employees in risk categories 3 and 4 were performed by four occupational health physicians (FL, PK, LM, FE — see Authors’ Contributions and Acknowledgements sections) using a standardized medical diagnostic procedure based on a criteria document [35, 36].

The prevalence of symptoms in the upper extremities, neck and lower back in the last 7-days and in the last 12-months, determined by the Nordic Questionnaire [37, 38], were considered as main outcomes of interest. The Nordic Questionnaire [37, 38] uses 5 categories (never, during last 1–7 days, …, always) to measure the prevalence of symptoms in the musculoskeletal system during the last 12 months. The categories of this variable were dichotomized (“never” = 0, all other categories = 1). The 7-day prevalence of symptoms was asked dichotomously.

Physical examinations were only performed for subjects in risk categories 3 and 4 (scale value ≥25 points). Due to the limited availability, physical examinations are handled as secondary outcomes of interest.

The sociodemographic and individual factors (age, body height, body mass index, working hours per week) were considered to control the regression analyses for potential sources of bias.

### Power calculation

An a-priory power calculation was done and published in the study protocol [34]. Assuming only a small difference in the prevalence of symptoms between exposed and unexposed subjects of nearly 0.25 (55 – 30%, corresponding to a prevalence ratio of 1.83), the power (1-beta) of the study was calculated for men as 99% (*n* = 650 unexposed, and *n* = 120 exposed men) and as 90% for women (*n* = 350 unexposed, and *n* = 80 exposed women).

### Statistical methods

Criterion validity of KIM-MHO was assessed by association between the risk category set by the scale value of KIM-MHO and the prevalence of symptoms in different regions of the musculoskeletal system.

Descriptive statistics (mean, standard deviations, absolute numbers, and percentage) were carried out according to STROBE (Strengthening The Reporting of OBservational studies in Epidemiology) [39].

Log-linear Poisson regression models with robust variance (IBM-SPSS-Statistics, GENLIN) estimation were applied. The natural exponent of the beta-coefficient (e^beta^) of log-linear regression models (Poisson regression) can be interpreted directly as prevalence ratio (PR) [40, 41]. Thus, the natural exponents of the effect estimates were calculated to obtain prevalence ratios for every risk category of KIM-MHO with 95%-confidence intervals (CI). Musculoskeletal symptoms during the last 7-days and the last 12-months in five body regions (neck/cervical spine, shoulder/upper arm, elbow/forearm, hand/wrist. and lower back) were used as outcomes in the regression models. All analyses were stratified by gender. All models were adjusted for age, body-mass-index and body height (numerical and centered by mean). Subjects were excluded from the regression analyses, if missing data occurred.

Based on the regression models and considering the intercept, post estimations of the prevalence of musculoskeletal symptoms with 95%-confidence intervals were calculated for each risk category and/or all subjects (mean prevalence). The adjusted mean prevalence obtained was centered for age, body-mass-index, and body height. For each outcome, linear effects across the four risk categories of KIM-MHO were tested to confirm dose-response relationships using the post-estimation option of the SPSS GENLIN function (/EMMEANS CONTRAST = POLYNOMIAL (1 2 3 4)).

Differences in the presence of clinical conditions in the upper extremities or the neck, based on the results of physical examinations, were analyzed similarly. Since physical examinations were only performed for subjects in risk categories 3 and 4 (≥ 25 points), subjects in risk category 3 (25 to 49 points according to KIM-MHO) were used as the reference group in the regression models and compared only to subjects in risk category 4.

## Results

### Descriptive data

The 642 employees (207 women, 435 men) exposed to MHO at 17 different work places were compared with a reference group of 804 employees (306 women, 498 men) working at VDT workstations. Both women and men in the study population were on average about 40 years old (women 39.9 +/- 10 years, men 39.7 +/- 9.2 years). Further characteristics of the study population regarding the number of subjects, age, body-mass index, body height and working hours per week are provided stratified by gender and by the four risk categories of KIM-MHO in Table 1.

**Table 1:** Distribution of study population characteristics

Parameter*	Risk category 1 (Reference) Employees in jobs with low MHO, < 10 points KIM-MHO	Risk category 2 Employees in jobs with MHO, 10 – 24 points KIM-MHO	Risk category 3 Employees in jobs with MHO, 25 – 49 points KIM-MHO	Risk category 4 Employees in jobs with MHO, ≥ 50 points KIM-MHO	All employees
Women	*N* = 306	*N* = 75	*N* = 59	*N* = 73	*N* = 513
age [years]	38.7 (10.1)	37.7 (8.9)	42.2 (9.1)	44.8 (9.9)	39.9 (10.0)
body height [cm]	167.6 (6.3)	169.2 (6.8)	164.7 (6.0)	164.7 (5.5)	167.1 (6.4)
body mass index	23.7 (3.6)	23.9 (4.3)	25.6 (4.7)	26.2 (4.9)	24.3 (4.2)
working hours per week [hours]	38.7 (6.6)	36.7 (5.6)	34.9 (3.1)	36.7 (3.1)	37.7 (5.9)
Men	*N* = 498	*N* = 173	*N* = 23	*N* = 239	*N* = 933
age [years]	41.5 (9.0)	38.5 (9.4)	39.7 (11.2)	36.6 (8.5)	39.7 (9.2)
body height [cm]	181.1 (6.8)	181.0 (6.9)	178.6 (6.3)	178.4 (6.8)	180.3 (6.9)
body mass index	26.1 (3.2)	26.2 (3.6)	25.5 (2.9)	26.8 (4.1)	26.3 (3.6)
working hours per week [hours]	43.3 (6.3)	40.0 (5.2)	38.5 (2.4)	37.7 (2.3)	41.1 (5.8)

*Distribution of different study characteristics by exposure to manual handling operations (MHO) according to KIM-MHO risk categories. Mean and standard deviation

The workplaces of the employees were analysed by the KIM-MHO and were assigned to “risk categories”. All VDT workplaces were assigned to risk category 1 (< 10 points; 306 women and 498 men), while the workplaces with MHO were assigned to the other three risk categories. In total, 75 women and 173 men were assigned to risk category 2, 59 women and 23 men to risk category 3, and 73 women and 239 men to risk category 4 (highest exposure group).

The percentage of missing values of the musculoskeletal symptoms in the different body region was below 1% for men and women. The percentage of missing values of confounder variables (age, body-mass index, and body height) was below 1% in men and women, except for the body mass index in women (2.7% missing values or 14 cases).

### Outcome data

Raw descriptions of the prevalence of symptoms during the last 7 days for different body regions stratified for exposed and unexposed subjects are provided in Table 2.

**Table 2:** Unadjusted (raw) prevalence of symptoms in the last 7 days

Body region by gender*	Risk category 1 (Reference) Employees in jobs with low MHO, < 10 points KIM-MHO	Risk category 2 Employees in jobs with MHO, 10 – 24 points KIM-MHO	Risk category 3 Employees in jobs with MHO, 25 – 49 points KIM-MHO	Risk category 4 Employees in jobs with MHO, ≥ 50 points KIM-MHO	All employees
Women	*N* = 306	*N* = 75	*N* = 59	*N* = 73	*N* = 513
neck/ cervical spine	90/304 (29.6%)	17/75 (22.7%)	27/59 (45.8%)	40/73 (54.8%)	174/511 (34.1%)
shoulder/ upper arm	58/304 (19.1%)	14/75 (18.7%)	23/59 (39.0%)	32/73 (43.8%)	127/511 (24.9%)
elbow/ forearm	13/304 (4.3%)	2/75 (2.7%)	11/59 (18.6%)	22/73 (30.1%)	48/511 (9.4%)
hand/ wrist	26/304 (8.6%)	4/75 (5.3%)	17/59 (28.8%)	34/73 (46.6%)	81/511 (15.9%)
lower back/lumbar spine	42/303 (13.9%)	8/75 (10.7%)	22/59 (37.3%)	24/73 (32.9%)	96/510 (18.8%)
Men	*N* = 498	*N* = 173	*N* = 23	*N* = 239	*N* = 933
neck/ cervical spine	86/498 (17.3%)	22/173 (12.7%)	4/23 (17.4%)	48/236 (20.3%)	160/930 (17.2%)
shoulder/ upper arm	70/498 (14.1%)	17/173 (9.8%)	7/23 (30.4%)	52/236 (22.0%)	146/930 (15.7%)
elbow/ forearm	28/498 (5.6%)	8/173 (4.6%)	3/23 (13.0%)	40/232 (17.2%)	79/926 (8.5%)
hand/ wrist	28/498 (5.6%)	11/173 (6.4%)	7/23 (30.4%)	72/234 (30.8%)	118/928 (12.7%)
lower back/lumbar spine	34/498 (6.8%)	19/171 (11.1%)	8/23 (34.8%)	85/238 (35.7%)	146/930 (15.7%)

*Unadjusted (raw) prevalence of symptoms in the last 7 days for different body regions by exposure to manual handling operations (MHO) according to KIM-MHO risk categories. Number of cases with symptoms/number of all available subjects (percentage of cases to all subjects)

The distribution of the estimated 7-day prevalence of symptoms in the musculoskeletal system in different body regions based on the regression analyses, taking into account age, BMI, and body height as confounders, is shown in Table 3. On the average, women reported symptoms of the neck/cervical spine and shoulder/upper arm more frequently than men. The 7-day prevalence increased with higher exposure as assessed by KIM-MHO. As an example, the estimated prevalence of symptoms in the last 7 days in the elbow region increased from nearly 5% in male subjects in the low (reference) and increased exposure groups to 12% and 17% in the highly increased and high exposure groups (risk categories 3 and 4 according to KIM-MHO).

**Table 3:** Estimated mean prevalence of symptoms in the last 7 days by exposure to MHO

Body regions by gender*	Risk category 1 (Reference) Employees in jobs with low MHO, < 10 points KIM-MHO	Risk category 2 Employees in jobs with MHO, 10 – 24 points KIM-MHO	Risk category 3 Employees in jobs with MHO, 25 – 49 points KIM-MHO	Risk category 4 Employees in jobs with MHO, ≥ 50 points KIM-MHO	All employees
Women	*n* = 306	*n* = 75	*n* = 59	*n* = 73	*n* = 513
neck/cervical spine	29.7% (24.9% – 35.4%)	23.9% (15.9% – 36.0%)	43.4% (32.4% – 58.1%)	52.0% (41.3% – 65.5%)	35.6% (30.7% – 41.2%)
shoulder/upper arm	19.9% (15.8% – 25.1%)	19.5% (12.4% – 30.9%)	35.6% (25.3% – 50.2%)	38.0% (28.4% – 50.8%)	27.0% (22.6% – 32.1%)
elbow/forearm	4.3% (2.5% – 7.4%)	2.7% (0.7% – 10.8%)	14.1% (7.9% – 25.0%)	19.3% (12.4% – 29.9%)	7.5% (5.0% – 11.3%)
hand/wrist	8.0% (5.4% – 11.8%)	4.2% (1.4% – 12.5%)	23.8% (15.5% – 36.7%)	33.7% (24.1% – 47.1%)	12.8% (9.3% – 17.7%)
lower back/lumbar spine	13.8% (10.4% – 18.4%)	11.3% (5.9% – 21.8%)	35.1% (24.6% – 50.2%)	30.6% (21.5% – 43.6%)	20.2% (16.2% – 25.2%)
Men	*n* = 498	*n* = 173	*n* = 23	*n* = 239	*n* = 933
neck/ cervical spine	16.5% (13.5% – 20.2%)	12.9% (8.7% – 19.0%)	16.7% (6.8% – 40.9%)	20.2% (15.5% – 26.4%)	16.4% (12.6% – 21.2%)
shoulder/upper arm	12.6% (9.9% – 15.9%)	9.8% (6.3% – 15.3%)	28.6% (15.5% – 53.0%)	23.6% (18.5% – 30.2%)	17.0% (13.8% – 21.0%)
elbow/forearm	5.1% (3.6% – 7.4%)	4.6% (2.4% – 8.8%)	12.1% (4.1% – 36.1%)	17.1% (12.5% – 23.4%)	8.3% (5.9% – 11.8%)
hand/wrist	5.5% (3.8% – 8.0%)	6.4% (3.6% – 11.3%)	30.8% (16.8% – 56.3%)	30.4% (24.6% – 37.7%)	13.5% (10.7% – 17.0%)
lower back/lumbar spine	6.8% (4.9% – 9.5%)	10.9% (7.1% – 16.6%)	33.4% (19.0% – 58.6%)	32.2% (26.2% – 39.7%)	16.8% (13.7% – 20.6%)

*Estimated mean prevalence of symptoms stratified by the exposure to manual handling operations (MHO) according to KIM-MHO risk categories. Based on regression models with 95%-CI, adjusted by age, body-mass-index and body height

**Table 4:** Unadjusted (raw) prevalence of symptoms in the last 12 months

Body region by gender*	Risk category 1 (Reference) Employees in jobs with low MHO, < 10 points KIM-MHO	Risk category 2 Employees in jobs with MHO, 10 – 24 points KIM-MHO	Risk category 3 Employees in jobs with MHO, 25 – 49 points KIM-MHO	Risk category 4 Employees in jobs with MHO, ≥ 50 points KIM-MHO	All employees
Women	*n* = 306	*n* = 75	*n* = 59	*n* = 73	*n* = 513
neck/cervical spine	201/304 (66.1%)	51/75 (68.0%)	50/59 (84.7%)	58/73 (79.5%)	360/511 (70.5%)
shoulder/upper arm	64/306 (20.9%)	13/75 (17.3%)	16/59 (27.1%)	24/73 (32.9%)	117/513 (22.8%)
elbow/forearm	42/306 (13.7%)	17/75 (22.7%)	18/59 (30.5%)	38/73 (52.1%)	115/513 (22.4%)
hand/wrist	78/306 (25.5%)	17/75 (22.7%)	29/59 (49.2%)	53/73 (72.6%)	177/513 (34.5%)
lower back/lumbar spine	107/303 (35.3%)	20/75 (26.7%)	42/59 (71.2%)	51/73 (69.9%)	220/510 (43.1%)
Men	*n* = 498	*n* = 173	*n* = 23	*n* = 239	*n* = 930
neck/cervical spine	244/498 (49.0%)	80/173 (46.2%)	18/23 (78.3%)	124/236 (52.5%)	466/930 (50.1%)
shoulder/upper arm	60/498 (12.0%)	29/173 (16.8%)	6/23 (26.1%)	54/236 (22.9%)	149/930 (16.0%)
elbow/forearm	72/498 (14.5%)	26/173 (15.0%)	4/23 (17.4%)	85/232 (36.6%)	187/926 (20.2%)
hand/wrist	91/498 (18.3%)	36/173 (20.8%)	11/23 (47.8%)	137/235 (58.3%)	275/929 (29.6%)
lower back/lumbar spine	117/498 (23.5%)	47/171 (27.5%)	15/22 (68.2%)	172/239 (72.0%)	351/930 (37.7%)

*Unadjusted (raw) prevalence of symptoms in the last 12 months for different body regions by exposure to manual handling operations (MHO) according to KIM-MHO risk categories. Number of cases with symptoms/number of all available subjects (percentage of cases to all subjects)

The distribution of the raw prevalence and the estimated adjusted prevalence of symptoms during the last 12 months is shown in Tables 4 and 5. The raw and estimated 12-months prevalence of symptoms is generally higher than the 7-day prevalence of symptoms for both genders and all outcomes. For example, taking confounders into account, the 12-months prevalence in the elbow region in men is estimated to be nearly 14% and 15% in the low (reference) and increased exposure groups, and 17% and 37% in the highly increased and high exposure groups (risk categories 3 and 4 of KIM-MHO). For further analysis, the adjusted effect estimates of the prevalence of symptoms were used to reduce potential confounding. In addition to the 7-day prevalence ratios (Table 6), the prevalence ratios for the occurrence of symptoms in the last 12 months are presented in Table 7.

**Table 5:** Estimated mean 12-months prevalence of symptoms by exposure to MHO

Body regions by gender*	Risk category 1 (Reference) Employees in jobs with low MHO, < 10 points KIM-MHO	Risk category 2 Employees in jobs with MHO, 10 – 24 points KIM-MHO	Risk category 3 Employees in jobs with MHO, 25 – 49 points KIM-MHO	Risk category 4 Employees in jobs with MHO, ≥ 50 points KIM-MHO	All employees
Women	*n* = 306	*n* = 75	*n* = 59	*n* = 73	*n* = 513
neck/cervical spine	66.3% (61.1% – 71.9%)	68.1% (58.0% – 80.0%)	84.2% (75.5% – 93.8%)	78.7% (69.3% – 89.4%)	74.0% (69.6% – 78.6%)
shoulder/upper arm	45.6% (40.3% – 51.7%)	53.2% (42.5% – 66.5%)	62.3% (51.4% – 75.5%)	57.1% (47.3% – 69.1%)	54.2% (49.3% – 59.6%)
elbow/forearm	13.5% (10.1% – 18.1%)	21.2% (13.4% – 33.5%)	26.0% (17.9% – 37.9%)	39.7% (30.3% – 52.0%)	23.3% (19.3% – 28.1%)
hand/wrist	25.0% (20.5% – 30.5%)	21.3% (13.7% – 33.3%)	46.1% (35.0% – 60.6%)	67.7% (57.0% – 80.5%)	35.9% (30.9% – 41.7%)
lower back/lumbar spine	34.3% (29.3% – 40.2%)	26.4% (18.1% – 38.6%)	69.3% (58.1% – 82.6%)	69.9% (59.3% – 82.3%)	45.7% (40.6% – 51.5%)
Men	*n* = 498	*n* = 173	*n* = 23	*n* = 239	*n* = 933
neck/ cervical spine	48.7% (44.5% – 53.4%)	46.7% (39.8% – 54.8%)	77.7% (62.7% – 96.5%)	52.2% (46.0% – 59.3%)	55.1% (51.0% – 59.6%)
shoulder/upper arm	32.2% (28.3% – 36.6%)	31.5% (25.4% – 39.2%)	65.1% (48.8% – 86.9%)	52.6% (46.2% – 59.9%)	43.2% (39.0% – 47.8%)
elbow/forearm	14.0% (11.2% – 17.3%)	15.2% (10.6% – 21.7%)	17.3% (6.9% – 43.3%)	37.1% (31.0% – 44.4%)	19.2% (14.9% – 24.8%)
hand/wrist	17.9% (14.8% – 21.7%)	20.7% (15.4% – 27.9%)	49.2% (32.2% – 75.0%)	58.1% (51.6% – 65.4%)	32.1% (27.9% – 36.9%)
lower back/lumbar spine	23.6% (20.1% – 27.7%)	27.4% (21.6% – 34.9%)	66.9% (50.0% – 89.4%)	68.6% (62.5% – 75.3%)	41.5% (37.4% – 46.1%)

*Estimated mean prevalence of symptoms in different body regions stratified by exposure to manual handling operations (MHO) according to KIM-MHO risk categories. [Prevalence, 95%-CI, adjusted by age, body-mass-index and body height]

**Table 6:** Prevalence Ratios (PR) of the 7-day prevalence of symptoms

Body regions by gender*	Risk category 1 (Reference) Employees in jobs with low MHO, < 10 points KIM-MHO	Risk category 2 Employees in jobs with MHO, 10 – 24 points KIM-MHO	Risk category 3 Employees in jobs with MHO, 25 – 49 points KIM-MHO	Risk category 4 Employees in jobs with MHO, ≥ 50 points KIM-MHO
Women	*n* = 306	*n* = 75	*n* = 59	*n* = 73
neck/cervical spine	1 (ref.)	0.8 (0.5 – 1.3)	**1.5 (1.0 – 2.1)**	**1.8 (1.3 – 2.3)**
shoulder/upper arm	1 (ref.)	1.0 (0.6 – 1.6)	**1.8 (1.2 – 2.7)**	**1.9 (1.3 – 2.8)**
elbow/forearm	1 (ref.)	0.6 (0.1 – 2.8)	**3.3 (1.5 – 7.2)**	**4.5 (2.3 – 8.7)**
hand/wrist	1 (ref.)	0.5 (0.2 – 1.7)	**3.0 (1.7 – 5.3)**	**4.2 (2.6 – 6.9)**
lower back/lumbar spine	1 (ref.)	0.8 (0.4 – 1.7)	**2.5 (1.6 – 4.0)**	**2.2 (1.4 – 3.5)**
Men	*n* = 498	*n* = 173	*n* = 23	*n* = 239
neck/cervical spine	1 (ref.)	0.8 (0.5 – 1.2)	1.0 (0.4 – 2.5)	1.2 (0.9 – 1.7)
shoulder/upper arm	1 (ref.)	0.8 (0.5 – 1.3)	**2.3 (1.2 – 4.4)**	**1.9 (1.3 – 2.7)**
elbow/forearm	1 (ref.)	0.9 (0.4 – 1.9)	2.4 (0.8 – 7.3)	**3.3 (2.1 – 5.4)**
hand/wrist	1 (ref.)	1.2 (0.6 – 2.3)	**5.5 (2.7 – 11.3)**	**5.5 (3.5 – 8.5)**
lower back/lumbar spine	1 (ref.)	1.6 (0.9 – 2.7)	**4.9 (2.5 – 9.4)**	**4.7 (3.1 – 7.1)**

*Prevalence Ratios of symptoms stratified by exposure to manual handling operations (MHO) according to KIM-MHO risk categories. Significant values are marked in bold letters

[Prevalence Ratios, 95%-CI. Log-linear Poisson regression, robust estimation of variance. One model per body region stratified by gender. Each model adjusted by age, body-mass-index and body height]

**Table 7:** Prevalence ratios (PR) of 12-months prevalence of symptoms

Body regions by gender*	Risk category 1 (Reference) Employees in jobs with low MHO, < 10 points KIM-MHO	Risk category 2 Employees in jobs with MHO, 10 – 24 points KIM-MHO	Risk category 3 Employees in jobs with MHO, 25 – 49 points KIM-MHO	Risk category 4 Employees in jobs with MHO, ≥ 50 points KIM-MHO
Women	*n* = 306	*n* = 75	*n* = 59	*n* = 73
neck/cervical spine	1 (ref.)	1.0 (0.9 – 1.2)	**1.3 (1.1 – 1.5)**	**1.2 (1.0 – 1.4)**
shoulder/upper arm	1 (ref.)	1.2 (0.9 – 1.5)	**1.4 (1.1 – 1.7)**	**1.3 (1.0 – 1.6)**
elbow/forearm	1 (ref.)	1.6 (0.9 – 2.7)	**1.9 (1.2 – 3.1)**	**2.9 (2.0 – 4.3)**
hand/wrist	1 (ref.)	0.9 (0.5 – 1.4)	**1.8 (1.3 – 2.6)**	**2.7 (2.1 – 3.5)**
lower back/lumbar spine	1 (ref.)	0.8 (0.5 – 1.2)	**2.0 (1.6 – 2.6)**	**2.0 (1.6 – 2.6)**
Men	*n* = 498	*n* = 173	*n* = 23	*n* = 239
neck/cervical spine	1 (ref.)	1.0 (0.8 – 1.2)	**1.6 (1.3 – 2.0)**	1.1 (0.9 – 1.3)
shoulder/upper arm	1 (ref.)	1.0 (0.8 – 1.3)	**2.0 (1.5 – 2.8)**	**1.6 (1.4 – 2.0)**
elbow/forearm	1 (ref.)	1.1 (0.7 – 1.6)	1.2 (0.5 – 3.2)	**2.7 (2.0 – 3.5)**
hand/wrist	1 (ref.)	1.2 (0.8 – 1.6)	**2.7 (1.7 – 4.4)**	**3.2 (2.6 – 4.1)**
lower back/lumbar spine	1 (ref.)	1.2 (0.9 – 1.6)	**2.8 (2.0 – 4.0)**	**2.9 (2.4 – 3.5)**

*Prevalence Ratios (PR) of symptoms in different body regions stratified by exposure to manual handling operations (MHO) according to KIM-MHO risk categories. Significant values are marked in bold letters. [Prevalence Ratios, 95%-CI. Log-linear Poisson regression, robust estimation of variance. Each model adjusted by age, body-mass-index and body height]

### Main results

Considering the Prevalence Ratios (PR) of musculoskeletal symptoms in correlation with the risk categories of the KIM-MHO, a dose-response relationship is visible, especially in the hand/wrist and elbow regions. The prevalence ratios for the occurrence of symptoms in the last 7 days is presented in Table 6. As an example, for women in the reference group (risk category 1) the adjusted 7-day prevalence of symptoms in the hand/wrist region is 8.0% (CI: 5.4–11.8%), but 23.8% (CI: 15.5–36.7%) and 33.7% (CI: 24.1–47.1%) for women with high exposures to manual handling operations (risk categories 3 and 4, see Table 3). In women, this corresponds to prevalence ratios of 3.0 (CI: 1.7–5.3) and 4.2 (CI: 2.6–6.9) in the highest risk categories (see Table 6). A similar increase in the prevalence of symptoms in the elbow region is shown in women with prevalence ratios of 3.3 (1.5–7.2) in risk category 3 and 4.5 (2.3–8.7) in risk category 4. In women, the prevalence ratios for symptoms in the last 7 days in the neck, shoulder and lower back regions are also significantly increased for exposed subjects in high exposure groups, but not to as great an extent as for symptoms in the elbow and hand/wrist regions.

In men, the 7-day prevalence is mainly increased for symptoms in the hand/wrist region. The prevalence ratios were 5.5 (CI: 2.7–11.3) and 5.5 (CI: 3.5–8.5) for the high exposure groups. For symptoms in the lower back the prevalence ratios of symptoms in the last 7 days were increased to a similar extent in the high exposure groups, but less increased in the elbow and shoulder regions. No increase of the prevalence ratios was observed regarding symptoms in the neck region (see Table 6). The 12-months prevalence in these joint regions show comparable increases for the high exposure groups (Table 7)﻿﻿.

**Fig. 1:**
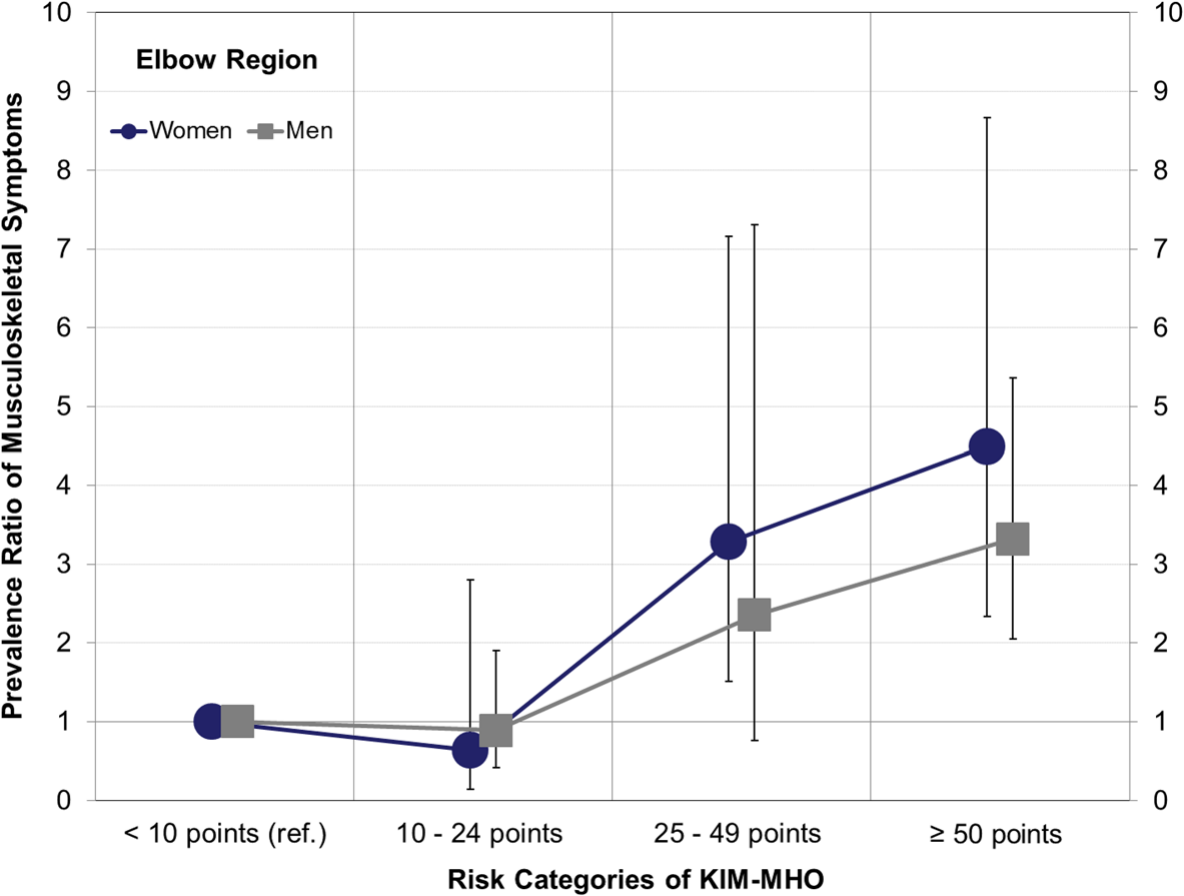
Prevalence ratios of the 7-day prevalence of elbow symptoms and exposure to manual operations

Figure 1 shows a typical dose-response relationship, a more or less linear relationship between the increasing intensity of manual handling operations and the increase in the prevalence ratios for the 7-day prevalence of symptoms of the elbow region for men and women. The prevalence of musculoskeletal symptoms is also increased in other regions (shoulder, neck, and lower back) in the high risk category 4, but without clear dose-response relationships and not to the same extent as symptoms in the hand/wrist or elbow regions. The post-estimations of linear trends based on the applied regression models confirm linear trends for all outcomes considered in men and women, excluding neck symptoms in men.

In the standardized physical examinations, women in risk category 4 had diagnoses of cervical spine syndrome (PR 1.78; 95%-CI 1.14–2.88) and carpal tunnel syndrome (PR 3.55; 95%-CI 1.31–2.19) more often than women in risk category 3. Among men, nonspecific musculoskeletal disorders (PR 13.0; 95%-CI 3–242) were observed more frequently in the high risk category 4 in KIM-MHO. In both genders, other diagnoses (such as rotator cuff syndrome, medial and lateral epicondylitis, Guyon canal syndrome) were also detected more often, but not increased significantly (data not shown).

## Discussion

The KIM-MHO was developed for the risk assessment of manual handling operations. This paper aimed to present the level of criterion validity. The KIM-MHO was also validated regarding additional quality criteria, including face validity, reliability and further aspects related to utility [33]. A cross-sectional study design was chosen to compare the workplace exposures, determined by work analyses and described by the risk category score of the KIM-MHO, to clinimetric parameters (MSDs as outcome criteria), determined by interviews and physical examinations of the employees. The relation of exposures and outcomes was assessed using log-linear Poisson regression models to provide adjusted prevalence ratios.

The results of the evaluation of the criterion validity support significant associations with a dose-response relationship between the level of exposure to MHO (KIM-MHO categories 3 and 4) and the frequency of musculoskeletal symptoms (predominantly in the hand/wrist and elbow/forearm regions, as well as in the shoulder region and a tendency of increase in the neck region). Physical examinations of the exposed employees showed a tendency of increase of relevant diagnoses in the upper extremities and neck regions in those employees with high exposures according to KIM-MHO scores.

In the present study, criterion validity was determined in a cross-sectional design. Therefore, it was only possible to show correlations between the predicted risk of prevalent adverse health outcomes by KIM-MHO at workplaces with MHO and the real prevalence of pre-existing musculoskeletal symptoms, but not in relation to incident adverse health events. In order to achieve a high-quality testing of methods, homogeneous job demands and employee groups in the occupational setting of manual work processes are necessary. However, homogeneous conditions are only rarely present in the work areas. Typically, order-related changes in work requirements and flexible staff assignments occur. This complicates the definition of the level of occupational exposure. Longitudinal studies are usually of higher value, such as in the validation of exposure assessment by HAL to predict carpal-tunnel syndrome [42–45]. However, in practice, a longitudinal method of looking at the possible link between occupational exposures and health related outcomes can hardly be realized, taking into account the necessity of accurate documentation of the changing occupational exposures during follow-up and the mostly recurrent characteristic of the health outcomes of interest. Since health related outcomes are individual and multifactorial, a direct association between working conditions and work-related symptoms is detectable only at relatively high exposures. These high exposures, however, were observed only at a few workplaces. While in epidemiological research the coherence of large groups of employees with similar workloads apply, the practical use of the KIM refers to specific working conditions. This basic contradiction must be considered. Also it has to be taken into account, that not all exposures automatically cause direct damage.

The aim of the present study was to determine the criterion-related validity by comparing the results of the KIM MHO with the prevalence of musculoskeletal symptoms in employees within a cross-sectional study design in terms of concurrent validity. The risk score provided by KIM-MHO can be interpreted as an external criterion. It provides hypotheses based on the concept, that higher values of the risk score are related to a higher probability of specific work-related adverse health outcomes. Considering the terminology of the COSMIN checklist (COnsensus-based Standards for the selection of health Measurement INstruments) [31], for evaluating the measurement properties of instruments, the validation of the risk score of KIM-MHO could also be seen as a part of the construct validity regarding the quality of hypotheses testing.

Prevalence of symptoms in the upper limbs region were used as the main outcome. Generally, the observed prevalence of symptoms in the upper extremities, neck and lower back for unexposed subjects of office workers is comparable to the prevalence in the German working population [46] and in other populations of office workers [47, 48]. Remembering earlier symptoms in a retrospective period could involve a recall bias. In order to minimize the bias and to get a more detailed overview, we asked for the past 7-days prevalence and the 12-months prevalence of symptoms. For most body regions, both 7-day and 12-months prevalences were associated with the exposure levels of the KIM. In addition, a physical examination was carried out, which found trends of an increasing number of diagnoses in the employees working at work places classified to the higher exposure levels of KIM-MHO. The observation of these three outcomes together might have reduced the recall bias.

The risk category of the KIM-MHO only describes the additional risk of MSDs according to the exposure to MHO. This does not mean, that employees in risk category 1 have no MSDs. As shown in Tables 2 and 4, there is already a base prevalence of symptoms in subjects with no or low exposure risk due to MHO. This high baseline prevalence of symptoms in the upper limbs and neck is seen in unexposed subjects as well [46–48]. This is especially true for the neck region, where the 12-months prevalence of symptoms in females in the no/low exposed risk category is already 66%. This might be the reason why no dose-response relationship could be described for higher risk categories due to MHO for the neck region.

The regression analyses consider age, gender, body mass index, and body height to reduce the risk of confounding by these risk factors. The incidence and prevalence of symptoms and diseases of the musculoskeletal system are age-dependent and differ between men and women [49, 50]. Body mass index is a surrogate for the risk factors overweight and obesity, and is correlated with individual muscle strength and fitness [51]. Body height reflects the differences in the individual working height and the physical workload to the upper extremities [52]. The bias caused by confounding is estimated as low considering the marginal differences between the raw and the adjusted effect estimates.

More than 40 observational tools have been developed to assess biomechanical exposures [28], but only some of these tools have been tested in a systematic manner for validity, repeatability, and aspects related to their practical use [26]. In their review, Takala et al. (2015) [26] identified 8 eligible observational methods addressing workload on upper limbs. Five of them were tested for associations with MSDs within cross-sectional studies. Among them, only two (Strain Index [24] and Hand Activity Level [25]) were tested additionally for prediction of MSDs in longitudinal studies. As far as comparable, the present validation of KIM-MHO shows a dose-response relationship in the same direction and dimension for exposures to MHO as in the validation studies mentioned above.

In regard to other forms of bias, it is important to emphasize that the unexposed subjects (office workers) and the exposed subjects were examined in different projects, but with the same methodology. We do not expect relevant effects from the time lag between the investigations. The participation of the employees in the study was voluntary. We do not have information about nonparticipants. The participation rate per workplace was high, but it is not possible to provide more detailed information about this. Selection bias cannot be excluded. If employees with complaints especially under exposure to MHO preferred to participate, the effects could be overestimated.

Nevertheless, from the perspective of the developers of the KIM, a broader view of the validity of the method is necessary. The criteria that determine whether the assessment results are correct (i.e. whether possible physical overuse is predicted accurately enough) may depend not only on the method itself. It is also of fundamental importance that valid input data are available. The experience gained from this project and the long-time experiences with the other KIMs repeatedly point to imprecise input data. Inaccurate time estimates, non-representative and superficial descriptions of exposures seem to be the most common faults. One conclusion is, therefore, that correct data assessment is imperative.

## Conclusions

The results of the evaluation process suggest satisfactory criterion validity of KIM-MHO, as the KIM risk score correlates significantly with the prevalence of MSDs in shoulder, elbow and hand/wrist regions and shows a dose-response relationship. Therefore, workplace risk assessment should use KIM-MHO in the case of exposures to MHO. If KIM-MHO indicates higher risks, measures of prevention such as job redesign, organizational changes, and provision of preventive occupational medical care should be considered. The usage of adverse health outcomes as parameters (clinimetric parameters) is useful, as long as the prevalence of symptoms in the reference group is low. If prevalence is high in the reference group (e.g. neck symptoms in office workers), only slight contrasts to high exposed groups of employees can be shown.

## Additional file

Additional file 1: The Key Indicator Method for the Risk Assessment of Manual Handling Operations (KIM-MHO). This document includes the worksheet of the KIM-MHO including a short manual, explaining how the assessment is to be performed. (PDF 242 kb)

## Abbreviations

ACGIH: American conference of governmental industrial hygienists
ART: Assessment of repetitive tasks of the upper limbs
BAuA: Bundesanstalt für Arbeitsschutz und Arbeitsmedizin [Federal Institute for Occupational Safety and Health]
BIBB: Bundesinstitut für Berufsbildung [Federal Institute for Vocational Education and Training]
BMI: Body mass index
CI: Confidence interval
COSMIN: COnsensus-based Standards for the selection of health Measurement INstruments
EEC: European Council Directive
GENLIN: Generalized linear models procedure
HARM: Hand-arm-risk-assessment method
IBM-SPSS-Statistics: Software package used for statistical analysis
KI: Key indicators
KIM: Key indicator method [Leitmerkmalmethode]
LHC: Lifting/holding/carrying
ManTRA: Manual tasks risk assessment tool
MHO: Manual handling operations
MSD: Musculoskeletal disorders
PP: Pulling/pushing
PR: Prevalence ratio
RC: Risk category
SI: Job strain index
TLV HAL: Threshold limit value for mono-tasks handwork
VDT: Visual display terminal
WRULD: Work related upper limb disorders
