# Supplementary material for: Risk assessment of manual handling operations at work with the key indicator method (KIM-MHO) — determination of criterion validity regarding the prevalence of musculoskeletal symptoms and clinical conditions within a cross-sectional study
Source: BMC Musculoskelet Disord. 2017 May 10;18:184. doi: 10.1186/s12891-017-1542-0 (PMC5424427; doi:10.1186/s12891-017-1542-0)
Supplement: Additional file 1: — The Key Indicator Method for the Risk Assessment of Manual Handling Operations (KIM-MHO). This document includes the worksheet of the KIM-MHO including a short manual, explaining how the assessment is to be performed. (PDF 242 kb) [file 12891_2017_1542_MOESM1_ESM.docx]

# Supplementary file: The Key Indicator Method for the Risk Assessment of Manual Handling Operations (KIM-MHO)

## Working procedure of KIM-MHO

How is the assessment by KIM-MHO performed?

- Fundamental procedure: The operation process (work cycle) must be observed carefully several times. Each arm/hand must be observed separately. We recommend at least 3 to 4 observations for each arm/hand or even more, if the operation process is complex. A video documentation is recommended to simplify observation analysis.
- A work cycle is taken to be a cohesive time phase in which a work process takes place.
- This may be a few seconds (e.g. inserting a part in a machine) or several minutes (e.g. complete assembly of a product). It is important that representative values are identified by counting and time measuring. Experience shows that for cycle times of up to 60 s an analysis of 5 to 10 cycles is sufficient. For larger cycle times, 10 to 15 cycles should be analyzed.
- It is important to know how many cycles the employee usually performs during the workday.
- To perform the assessment, the following steps as indicated in Figure 1 should be executed:
- The number of work cycles per day multiplied by the cycle-time indicates the daily duration of manual work processes [key indicator (KI 1)].
- For the assessment of the type, the duration and the frequency of force exertion(s) (KI 2), the total frequencies of moves must be counted and/or the total duration of holding must be measured. These values are converted to a “standard minute”, if cycle time is unequal to one minute. In this case, the values are divided by the number of minutes observed. From this, it is possible to calculate the average holding times and average movement frequencies during one standard minute.
- Further aspects to be observed are the force transmission and gripping conditions (KI 3), the main hand-arm postures during manual work processes (KI 4), the dominating body posture during manual work processes (KI 5), the work organization (KI 6), and the working conditions (KI 7).

## Figure 2: Procedure and steps of workplace assessment with KIM-MHO


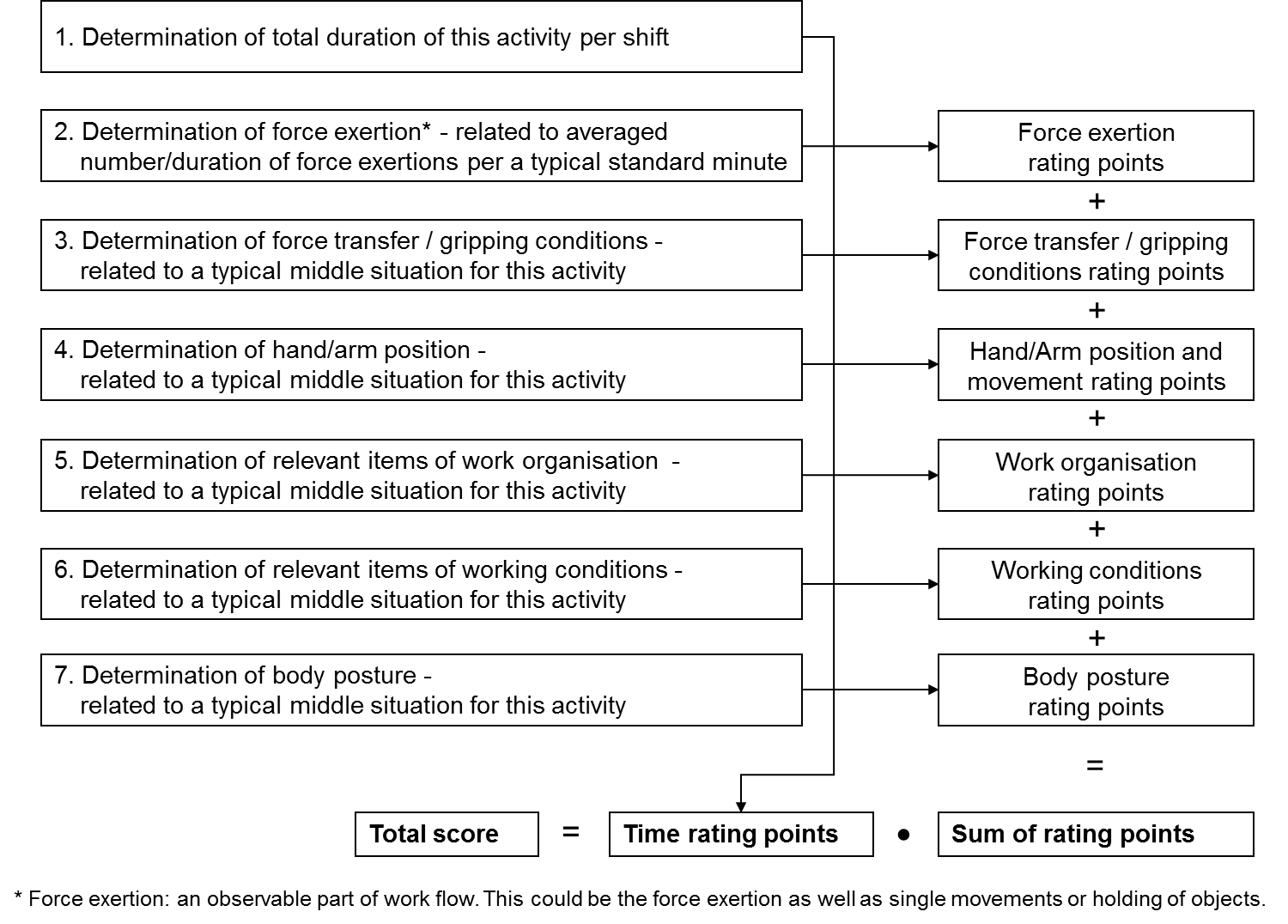


## Form of KIM-MHO

| **Key indicator method for assessing physical workload during manual handling operations** | | |
| --- | --- | --- |
| If a number of different tasks are performed within one working day, they must be recorded separately. | | |
| **Description of task:** |  | **V2012** |
|  | | |
| ***1^st^ step: Determination of time rating points*** | | |
| \| **Total duration of this activity per shift**  [up to … hours] \| 1 \| 2 \| 3 \| 4 \| 5 \| 6 \| 7 \| 8 \| 9 \| 10 \| \| --- \| --- \| --- \| --- \| --- \| --- \| --- \| --- \| --- \| --- \| --- \| \| **Time rating points** \| **1** \| **1.5** \| **2** \| **2.5** \| **3** \| **3.5** \| **4** \| **4.5** \| **5** \| **5.5** \| | | |
|  | | |
| ***2^nd^ step: Determination of the rating points for the type of force exertion, force transfer/gripping conditions, hand/arm positions and movement, work organisation, working conditions, and body posture*** | | |
| \| **Type of force exertion(s)  in the finger-hand area** \| \|  \| **Holding** \| \| \| \| **Moving** \| \| \| \| \| \| \| \| \| --- \| --- \| --- \| --- \| --- \| --- \| --- \| --- \| --- \| --- \| --- \| --- \| --- \| --- \| --- \| \| **average holding time**  **[seconds per minute]** \| \| \| \| **average movement frequencies**  **[number per minute]** \| \| \| \| \| \| \| \| \|  \| \|  \| **60-31** \| **30-16** \| **15-4** \| **<4** \| **<1** \| **1-4** \| **5-15** \| **16-30** \| \| **31-60** \| \| **>60** \| \| **Level** \| **Description, typical examples** \| **Rating points** \| \| \| \| \| \| \| \| \| \| \| \| \| low  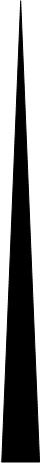  high \| **very low forces** e.g. keyboard or button control operations / shifting / arranging \| **2** \| **1** \| **0.5** \| **0** \| \| **0** \| **0.5** \| **1** \| \| **2** \| \| **3** \| \| **low forces** e.g. guiding material / inserting small objects \| **3** \| **1.5** \| **1** \| **0** \| \| **0** \| **1** \| **1.5** \| \| **3** \| \| **5** \| \| **moderate forces**  e.g. gripping / assembling / putting together small work pieces by hand or with small tools \| **5** \| **2** \| **1** \| **0** \| \| **0,5** \| **1** \| **2** \| \| **5** \| \| **8** \| \| **high forces**  e.g. turning-twisting / wrapping-packaging / grasping / holding or joining together work pieces, pressing them together / cutting / working with small powered hand tools \| **8** \| **4** \| **2** \| **0.5** \| \| **1** \| **2** \| **4** \| \| **8** \| \| **13** \| \| **very high forces** e.g. cutting involving considerable force / working with tools such as small staple guns / moving or holding parts or larger tools \| **12** \| **6** \| **3** \| **1** \| \| **1** \| **3** \| **6** \| \| **12** \| \| **21** \| \| **peak forces** e.g. forceful screwing / loosening bolts / pressing in \| **19** \| **9** \| **4** \| **1** \| \| **2** \| **4** \| **9** \| \| **19** \| \| **33** \| \| **hitting** with ball of the thumb, palm of hand or fist \| **-** \| **-** \| **-** \| **1** \| \| **1** \| **3** \| **6** \| \| **12** \| \| **21** \| \| *The work cycle must be observed and the rating points for the force categories marked. Added together (left and right hands separately) these yield the force rating points. To calculate the total point rating values, the higher value is used.* \| \|  \| **Rating points of force exertion:** \| \| \| \| \| \| \| \| **Left hand:** \| \| **Right hand:** \| \| | | |
|  | | |
| \| **Force transfer / Gripping conditions** \| **Rating points** \| \| --- \| --- \| \| **Optimum force transfer/application /** working objects are easy to grip  (e.g. bar shaped, gripping grooves) / good ergonomic gripping design (grips, buttons, tools) \| **0** \| \| **Restricted force transfer/application** / greater holding forces required / no specially shaped grips \| **2** \| \| **Force transfer / application considerably hindered** / working objects hardly possible to grip  (slippery, greasy, soft, sharp edges) / no or unsuitable grips \| **4** \| | | |
|  | | |
| \| **Hand/Arm position and movement ^*)^** \| \| **Rating points** \| \| --- \| --- \| --- \| \| 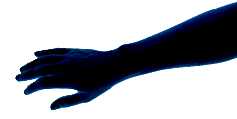 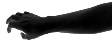 \| **Good:** position or movements of the joints are in the medium (relaxed) range /  only rare deviations \| **0** \| \| 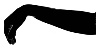 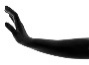  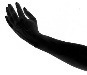 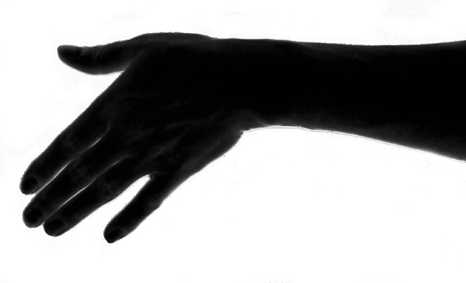  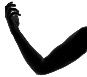 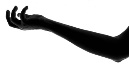 \| **Restricted:** occasional positions or movements of the joints at range-of-motion limits \| **1** \| \| **Unfavourable:** frequent positions or movements of the joints at range-of-motion limits \| **2** \| \| **Poor:** constant positions or movements of the joints at range-of-motion limits / prolonged static holding of the arms without hand-arm support \| **3** \| \| *^*)^ Typical positions are to be considered. Rare deviations can be ignored.* \| \| \| | | |
|  | | |
|  | | |
| \| **Work organisation** \| **Rating points** \| \| --- \| --- \| \| **Frequent variation of exposure/load situation** due to other activities / several different work operations /  sufficient opportunity for recuperation \| **0** \| \| **Infrequent variation of exposure/load situation** due to other activities / few different work operations /  recuperation times adequate \| **1** \| \| **Poor / almost no variation of exposure/load situation** due to other activities / few single movements per work operation / high work rate due to high line balancing and/or high piece work output / unbalanced work flow with concurrent high exposure peaks of strain / too few or too short recuperation times \| **2** \| \| *Elements not mentioned in the table are to be taken into account accordingly.* \| \| | | |
|  | | |
| \| **Working conditions** \| **Rating points** \| \| --- \| --- \| \| **Good:** reliable recognition of details / no glare / good climatic conditions \| **0** \| \| **Restricted:** impaired detail recognition due to glare or excessively small parts / air draughts / cold temperatures / wet conditions / dirt / disturbance of concentration due to noise \| **1** \| \| *Elements not mentioned in the table are to be taken into account accordingly. In case of highly unfavourable conditions, rating point 2 can be assigned.* \| \| | | |
|  | | |
| \| **Body posture ^**)^** \| \| **Rating points** \| \| --- \| --- \| --- \| \| 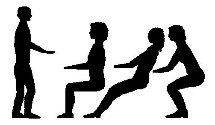 \| **Good:** alternation of sitting and standing is possible / alternation of standing and walking is possible / dynamic sitting is possible / hand-arm rest is possible if required / no twisting / head posture variable / no gripping above shoulder height \| **0** \| \| 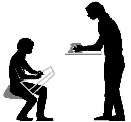 \| **Restricted:** trunk with slight inclination of the body towards the area of action /  predominant sitting with occasional standing or walking / occasional gripping above shoulder height \| **1** \| \| 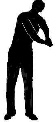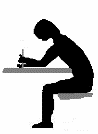 \| **Unfavourable:** trunk clearly inclined forward and/or twisted / head posture specified for detail recognition / restricted freedom of movement / constant standing without walking / frequent gripping above shoulder height / frequent gripping at a greater distance from the body \| **3** \| \| 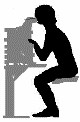 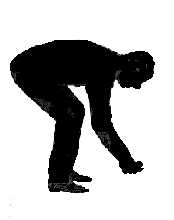 \| **Poor:** trunk severely twisted and inclined forward / body posture strictly fixed /  visual check of action through magnifying glasses or microscopes / severe inclination or twisting of the head / frequent bending / constant gripping above shoulder height / constant gripping at a greater distance from the body \| **5** \| \| *^**)^ Typical postures are to be taken into account. Rare deviations can be ignored.* \| \| \| | | |
|  | | |
| ***3^rd^ step: Evaluation*** | | |
| Enter the rating points applicable for the activities and calculate the risk score in the diagram. | | |
|  | | |
| \|  \| Finger-Hand area force exertion rating points \|  \|  \|  \| \| \| \| --- \| --- \| --- \| --- \| --- \| --- \| --- \| \| + \| Force transfer/gripping conditions rating points \|  \| \| + \| Hand/Arm position and movement rating points \|  \| \| + \| Work organisation rating points \|  \| \| + \| Working conditions rating points \|  \| \| + \| Body posture rating points \|  \| \| **=** \| **Total:** \|  \| **x** \| Time rating points \| **=** \| Risk score \| \|  \| | | |
|  | | |
| On the basis of the risk score calculated and the table below it is possible to make a rough evaluation. | | |
| \| **Risk range** ***^)^ \| \| **Risk score** \| **Description** \| \| --- \| --- \| --- \| --- \| \| 1 \| 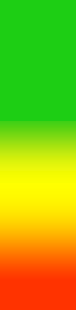 \| < 10 \| Low exposure situation. Physical overload due to MHO unlikely. \| \| 2 \| 10 to < 25 \| Increased exposure situation. Physical overload due to MHO possible for particular groups of employees. Redesign of the workplace might be helpful for this group. \| \| 3 \| 25 to < 50 \| Highly increased exposure situation. Physical overload due to MHO possible.  Redesign of the workplace is recommended. \| \| 4 \| ≥ 50 \| High exposure situation. Physical overload due to MHO likely.  Redesign of the workplace is necessary. \|   ^***)^ The boundaries between the categories of risk ranges are fluid because of individual differences in working techniques and performance conditions. The classification should therefore only be regarded as an **orientation aid**. Basically it must be assumed that as the risk scores rises, the risk of overload of the musculoskeletal system increases. | | |
| Published by the Federal Institute for Occupational Safety and Health / [www.baua.de](http://www.baua.de) | | |
